# Exploring service providers’ perspectives on the prevention and management of fetal alcohol spectrum disorders in South Africa: a qualitative study

**DOI:** 10.1186/s12889-018-6126-x

**Published:** 2018-11-06

**Authors:** Babatope O. Adebiyi, Ferdinand C. Mukumbang, Lizahn G. Cloete, Anna-Marie Beytell

**Affiliations:** 1 School of Public Health, University of the Western Cape, Robert Sobukwe Road, Bellville, Cape Town, 7535 South Africa; 2 Department of Social Work, University of the Western Cape, Cape Town, South Africa; 3 Division of Occupational Therapy, University of Stellenbosch, Stellenbosch, South Africa

**Keywords:** Policies, Guidelines, Fetal alcohol Spectrum disorders, Service providers, Interventions, Services, Women, Development disabilities, Prevention, Management

## Abstract

**Background:**

Fetal alcohol spectrum disorder (FASD) is among the leading causes of developmental and intellectual disabilities in individuals. Although efforts are being made toward the prevention and management of FASD in South Africa, the prevalence remains high. The sustained high prevalence could be attributed to several factors, including the lack of policy for a coordinated effort to prevent, diagnose and manage FASD nationally. In this study, our aim was to explore the perspectives of service providers (health and allied professionals, teachers, social workers) on the prevention and management of FASD towards developing a guideline to inform policy.

**Method:**

Guided by the exploratory qualitative research design, we purposively sampled relevant service providers in the field of FASD prevention and management for focus group discussions. Nine of these discussions were conducted with to eight participants per discussion session. The discussants were asked various questions on the current and required interventions and practices for the prevention and management of FASD. Following the Framework Method, data were transcribed verbatim and analysed using the thematic content analysis approach.

**Results:**

Our findings show that aspects of the prevention and management of alcohol-related conditions are present in various policies. However, there is no clear focus on coordinated, multi-sectoral efforts for a more comprehensive approach to the prevention and management of FASD. The participants recognized the need for specific requirements on broad-based preventive awareness programs, training and support for parents and caregivers, inclusive education in mainstream schools and training of relevant professionals.

**Conclusion:**

Comprehensive and coordinated prevention and management programs guided by a specific policy could improve the prevention and management of FASD. Policy formulation demonstrates commitment from the government, highlights the importance of the condition, and elaborates on context-specific prevention and management protocols.

**Electronic supplementary material:**

The online version of this article (10.1186/s12889-018-6126-x) contains supplementary material, which is available to authorized users.

## Background

Although generic programs and interventions exist for the prevention and management of fetal alcohol spectrum disorder (FASD), the prevalence in South Africa continues to rise. FASD is a group of conditions used to describe a range of developmental disabilities resulting from prenatal alcohol exposure [1–3]. Individuals with FASD may have abnormal facial features, low body weight, poor coordination, learning disabilities, difficulty with attention, poor reasoning and judgment skills, speech and language delays, hyperactive behavior, and intellectual difficulties or a low IQ (intellectual quotient) [4].

The global prevalence of FASD has been estimated at 8 per 1000 children and youth in the general population [5]. Zelner and Koren [6] estimated the prevalence of FASD at 9 per 1000 live births in Canada. In Italy, the prevalence of FASD was reported to be 23 to 47 per 1000 in first-grade pupils [7], while in Croatia, it was estimated to be 40 per 1000 in elementary school children [8]. In four communities of USA, the prevalence was estimated to be 11 to 50 per 1000 children [9], and in a secluded Indigenous population in Australia the reported prevalence was 194.4 per 1000 children [10].

In South Africa, FASD prevalence has been estimated to be between 29 to 290 per 1000 live births [11]. In the Western Cape Province, studies conducted on the prevalence of FASD among primary school pupils showed a persistent increase over the years. In 2007, the prevalence of FASD was estimated at 89.2 per 1000 [12], and by 2013, it had doubled (135.1 to 207.5 per 1000) [13]. In 2016, the prevalence of FASD showed a persistent increase (170 to 233 per 1000) [14], and in 2017 yet another increase among the same group (196 to 276 per 1000) was recorded [15]. Owing to excessive alcohol use, the Northern and Western Cape Provinces carry the brunt of FASD in South Africa [16, 17]. The persistent rise in the FASD prevalence in these two Provinces highlights the need for a coordinated effort – context-specific preventive interventions, early identification, diagnosis, and management – in dealing with FASD.

FASD is recognized as a public health concern in most countries around the world [18–20]. Public health practitioners have advocated for specialized services to address FASD [18, 21]. Services such as context-specific prevention programs, well-resourced mental health facilities for the management of FASD-related mental health problems, specialized schools to manage learning disabilities, and alcohol rehabilitation services for people with substance abuse problems have been identified as pivotal to the prevention and management of FASD [18]. In addition, community-based programs to support pregnant women and sustainable commitment from various governments and service providers are required to address the high FASD prevalence [18, 21]. Furthermore, Poole et al. [22] identified a four-level comprehensive approach to the prevention and management of FASD, with level two focusing on women of reproductive age and their partners. This comprehensive approach is a step closer to a social determinant approach, which seeks to understand the social, historical, political, and economic underlying causes of disease.

Policy development for FASD aligns with goals three and four of the Sustainable Development Goals [23]. For interventions and programs to be effective and sustainable, they should be embedded in guidelines that may facilitate the development of a FASD policy [24, 25]. Policy development is a step towards designing legislations, which would allow practitioners to make informed decisions, take prompt treatment and management actions, and plan, develop and implement comprehensive community-based interventions [24, 26]. While developing a policy may not be a panacea to achieving the effectiveness and sustainability of interventions for FASD, it may create a framework for the implementation and support for individualized and integrated care [25, 27]. Countries like Canada and Australia with FASD problems similar to South Africa have acknowledged the importance of policies for addressing FASD [28–30]. In South Africa, however, there are no specific FASD policies [31].

Despite the absence of a specific FASD policy [20, 32], other health-related policies contain clauses that could be attributed to the prevention and mangement of FASD in South Africa. These policies include the Prevention and Treatment of Drug Dependency Act, South Africa Social Security Act, Children’s Act 38 of 2005, National Programme of Action for Children, Human Genetics Policy Guidelines for the Management and Prevention of Genetic Disorders, Birth Defects and Disabilities. Other documents include the National Guidelines for the Management and Prevention of Drug Use and Abuse, National Drug Master Plan 2006–2011, Guidelines for Maternity Care in South Africa, Education White Paper 5 on Early Childhood Development and Education White Paper 6 on Inclusive Education [31]. In addition, an American-based practical clinical approach to the diagnosis of FASD based on the institute of medicine's criteria developed by USA researchers has been used to diagnose FASD in South Africa [33].

Rendall-Mkosi et al. [31] suggested a comprehensive FASD prevention and management approach to be adopted by various government departments (Table 1). Some of the services identified in their approach to prevention and management of FASD were also mentioned in the Australian action plan on FASD [30] and the Canadian framework for action on FASD [34]. In the Australian action plan, the proposed principles for action were population health framework, a whole government approach, human rights-based approach, and women-centered practice. The Canadian framework for action on FASD also highlighted that a comprehensive approach to prevention and management of FASD requires individual and collaborative actions, all sectors, and all government levels (national, provincial and community). In addition, efforts need to focus on prevention, meeting current needs of people with FASD, and strengthening and expanding the support systems, services, and resources.

**Table 1:** Relevant government departments’ responsibilities towards the prevention and management of FASD

Relevant government departments	Responsibilities towards prevention of FASD	Responsibilities towards the management of FASD
Department of Health	– Estimate prevalence of FAS and FASD – Determine surveillance strategy for Alcohol-exposed pregnancy (AEP) – Screen for women at high risk for an AEP: binge drinking without using contraceptives – Implement strategies to prevent AEP in high-risk settings e.g. brief interventions at primary level – Create and disseminate educational materials on medical issues for FASD to the public and service providers – Training of service providers on medical issues for FASD	– Developmental screening and referral at birth and infancy. – Diagnosis, feeding support and parenting support in early childhood. – Contraception services for individuals with FASD – Mental health services for individuals with FASD
Department of Education	– Provide secondary and tertiary level education for adolescents and young women – Life skills training in secondary education – Create and disseminate educational materials on educational issues for FASDs to the public and service providers – Training of service providers on educational issues for FASD	– Provide crèches and pre-schools for individuals with FASD – Teacher and parent training on how to manage learning, language, and attention problems in individuals with FASD – Supportive educational environment for individuals with FASD – Classroom support for teachers – Development of specific learning interventions – Life skills training for individuals with FASD
Department of Social Development	– Support for women at high alcohol risk and their families. – Recreational activities to promote healthy life styles – Access to specialised alcohol rehabilitation services – Create and disseminate educational materials on social issues for FASD to the public and service providers – Training of service providers on social issues for FASD	– Access to social grants. – Family support against stigma and assistance with child management in the family. – Developing supportive home environment for women and individuals with FASD – Supportive community for individuals with FASD – Promoting independence among individuals with FASD
Department of Justice, Safety, and Security	– Enforcing liquor laws – Regulation of liquor industry – Legal protection for women and individuals with FASD – Create and disseminate educational materials on legal issues for FASD to the public and service providers – Training of service providers on legal issues for FASD	– Vulnerable to physical and sexual abuse and domestic violence – Better awareness by the police of FASD vulnerability e.g. recognition of disability in the legal process
Department of Labour	– Work-related skills training for Individuals with FASD – Employment opportunities for women – Create and disseminate public and provider educational materials on labour issue for FASD to the public and service providers –Training of service providers on labour issues for FASD	– Maternity leave for women to ensure early proper management for individuals with FASD – Efficient unemployment insurance fund (UIF) process – Alternative skills training for individual with FASD – Meaningful employment opportunities for individual with FASD (sheltered/menial work)

To incorporate the above-mentioned approach and principles and address the lack of a coordinated and holistic plan to tackle FASD in South Africa, we propose the development of a guideline to inform a multi-sectoral and interdepartmental policy [35]. In this paper, we report on a step toward developing a guideline for policy on FASD – exploring the perspectives of teachers, social workers, occupational therapists, nurses, pediatricians, doctors, and psychologists on the prevention and management of FASD in South Africa.

## Methods

### Study setting

The study was conducted in the Western Cape Province of South Africa. The Western Cape Province is the fourth largest and the third most populated Province in South Africa with an estimated 6.5 million residents [36].

In the Western Cape Province, alcohol-related business activities ranked second in the most common category of business in the township economy [37]. Most of these businesses are run by women to complement their family income. There are about 25,000 illegal liquor stores (known as shebeens - home-based taverns) in the province, making alcohol accessible to people of all ages [37]. This province is also known for its historical ‘Dop’ system whereby farm workers’ partial wages were paid in the form of alcoholic beverages [38]. The legacy of the ‘Dop’ system reflects a distinctive drinking culture deep-rooted in the social matrix of farm workers as well as informal settlements where employers paid their laborers with cheap wine [38]. Alcohol abuse is, therefore, rife in the Western Cape Province owing to its cultural context [39].

According to Statistics South Africa [40], in the Western Cape, 38.1% of women reported consuming alcohol at least once during their lifetime and 27.3% reported drinking alcohol in the past 12 months. In addition, 18.0% reported drinking alcohol in the past seven days and 9.0% reported consuming five or more drinks on at least one occasion in the past 30 days. Furthermore, according to Petersen Williams et al. [41], 36.9% of women in the Western Cape Province consumed alcohol during pregnancy or in the last three months before they were aware of their pregnancy. The uncontrolled sale and drinking patterns displayed by the community, including women who are pregnant reveal the endemic nature of the risk factors for FASD in the Western Cape [42] and could be the reason behind the increasing prevalence of FASD [14].

### Study design

We employed an exploratory qualitative study design to investigate the perspectives of the service providers. According to Glenton, Lewin, Norris and Norris [43], evidence from qualitative research enriches the development and implementation of guidelines as it permits a comprehensive exploration of the perspectives and considerations of relevant stakeholders.

### Sampling procedure

For FASD to be effectively prevented, identified, diagnosed, and managed, a wide range of professionals is required. These professionals should consist of nurses, social workers, physicians, psychologist, occupational therapist, speech-language pathologist, and primary healthcare providers (who provide antenatal care and counselling for pregnant women and preventive services for community members) [42]. The professionals may also include addiction counsellors, childcare workers, cultural interpreters, mental health workers, parents or caregivers, probation officers, psychiatrists, teachers, vocational counsellors, geneticists or dysmorphologists, neuropsychologists, and family therapists [2, 28, 29].

A two-step purposive sampling approach was employed to select the relevant institutions (healthcare facilities, schools, and [non-profit organizations] NPOs) and service providers (allied health, medical, nurse, teacher, and other professionals). In the first step, after permission was obtained from the study host University, we approached each department (Education, Health, and Social Development) for permission. Thereafter, we purposively selected 13 institutions because they render prevention or/and management services on FASD in the Western Cape to participate in the study. However, only ten facilities participated, which included four healthcare facilities, three schools, and three NPOs. In step two, we selected the study participants from the selected institutions. We sought to include service providers with at least 5 years of experience in service delivery and implementing prevention interventions for women or management interventions for individuals with FASD that were members of a multidisciplinary team. In Table 2, the characteristics of the 65 participants that were included in the study are shown.

**Table 2:** Characteristics of the study participants

Characteristics	Participants (*N* = 65)
Types of institutions	
Schools	3
Healthcare facilities	4
NPOs	2
Gender	
Male	5
Female	60
Profession	
Allied health	20
Medical	10
Nurse	6
Teacher	26
Others	3
Working experience (years)	
1–10	33
11–20	19
21–30	10
31–40	3

### Data collection

Focus group discussions (FGDs) were conducted in English between September 2016 and September 2017. The discussions were guided by a discussion schedule (Additional file 1). Open-ended questions were used to start the discussions and we used follow-up questions to probe for additional explanations from the participants when necessary. The study participants were asked various questions on policies that guide the FASD interventions and services, and relevant aspects of FASD to inform the FASD policy. Each discussion lasted about 30–60 min and was audio-recorded with permission from the participants. Nine FGDs were conducted with six to eight participants in each group. While conducting the FGDs multiple participants were asked questions, and their responses were noted [44]. Our data collection process was guided by data saturation [44, 45]. We reached data saturation when new information was not elicited with subsequent discussions, and we judged that there were enough data to answer the research questions [46, 47].

### Data analysis

The Framework Method was used to analyze the data [48]. Framework Method is part of the thematic analysis family [49]. Using the Framework Method, one author (BOA) transcribed the discussions, and two of the authors (BOA, FCM) read these thoroughly for familiarization. Notes and memos were made from the transcripts to inductively generate the initial codes, and these codes were re-organized to obtain refined codes. Using the initial codes, two of the authors (BOA, FCM) developed a coding guide (Appendix 1) and discussed this with the research team (BOA, FCM, LGC, AMB). Codes with similar ideas were clustered to form sub-themes and sub-themes with identical concepts were further grouped to form the final themes. The coding guide developed was used by ascribing relevant aspects of the texts in the subsequent transcripts to existing categories, themes, and sub-themes. Finally, we charted the data into the framework matrix as illustrated in Additional files 2 and 3.

### Trustworthiness and rigor of the study

In this study, rigor and trustworthiness were established through explicit measures to ensure credibility, transferability, dependability, conformability, and through a reflexive approach to the inquiry and analysis [50]. To ensure credibility, during the data collection, we ensured that the discussants were allowed to express themselves freely during the discussions. At the end of each FGD, we revisited the main points that came out of the discussions to confirm agreements, disagreements, additions, and corrections from the participants. We also kept a reflective journal during the study, which documented the discussions, deliberations, and decisions of the research team. To ensure the credibility of the study findings, we provided a comprehensive description of the study methodology, and the findings are supported by verbatim responses from the participants. In reporting this study, we followed the relevant aspects of the consolidated criteria for reporting qualitative research (COREQ) [51] (Appendix 2). The discussion schedule was designed based on FASD literature and in consultation with the research team to ensure dependability. We also ensured the use of the same discussion schedule to guide all the discussions. Furthermore, one member of the research team (BOA) under the guidance of another member (FCM) coded the transcriptions and met afterward to discuss the findings.

### Data management

To maintain anonymity and easy identification of the information sources, we coded service providers (health professionals, teachers, and NPO workers) in the various departments (Education, Health, and Social Development) as DOE_X_, DOH_X_, and DSD_X_. The _X_ denotes an arbitrary number from 1 to 12. The information will be kept for 10 years.

## Results

Following the coding guide (Appendix 1) the findings are presented under the categories, availability of guidelines/policies on FASD, development of guideline/policy document, current practices and available interventions, and identified policy requirements for FASD.

### Category 1: No available guidelines/policies on FASD

All the selected relevant service providers (health professionals, teachers, and NPO workers) acknowledged the absence of policies/guidelines to coordinate efforts toward addressing the issues around FASD.

There is no policy that I am aware of, either in the hospital or at the [Western Cape] province as far as FASD are concerned. **(DOH**_**2**_ **Health professional**_**8**_**)**

There is no guideline to guide us on how to do things, how to deliver our services better than what we are doing now. I think the research [on FASD] is not sufficient to guide the policy makers at the moment [to develop policy on FASD]. **(DSD**_**2**_ **NPO worker**_**3**_**)**

There is no clear policy or guideline for us that guides the intervention. Our NPO is under the Department of Social Development (DSD) and with our intervention programs, we have just started prioritizing [FASD] and I think that it is why your study is so important to see what [FASD] policies that can be put in place. **(DSD**_**1**_ **NPO worker**_**3**_**)**

Some of the participants, however, indicated that various clauses exist in different policies, which could be used to guide the prevention and management of FASD.

No, we do not [have a policy for FASD], they have mentioned [FASD clauses] in health [polices], but there is no clear [FASD] policy. **(DSD**_**1**_ **NPO worker**_**1**_**)**

I think the government has it [FASD-related management] on add-on basis [in other policies]. [For example], the Department of Education has a policy on inclusive education that will govern the education of all children with special needs, including children with FASD. The Department of Health recognized these [FASD] as a mental health issue and they [FASD] are mentioned in Western Cape Government policy like first 1,000 days. I do not think there is a specific document called FASD policy in Western Cape, but you can find an overview of FASD in different policies. **(DSD**_**1**_ **NPO worker**_**2**_**)**

### Category 2: The need for developing a guideline/policy document

Some participants expressed the need for a separate guideline/policy to encourage a holistic and robust approach to address FASD issues. The participants expressed the need for a document that will specify clearly the needs of individuals with FASD.

Our non-profit organization is under the DSD and with our intervention programs, we have just started prioritizing [FASD] and I think that it is why your study is so important to see what [FASD] policies that can be put in place. **(DSD**_**1**_ **NPO worker**_**3**_**)**

We need a document that will state these are the needs of the children with [FASD]. There must be a clear plan, for example, if a child [with an FASD] enters into a system, what are the needs of that child [with an FASD]. This must be in a document. [This is] because from this document, the NPOs can start creating services that are necessary. **(DSD**_**1**_ **NPO worker**_**3**_**)**

The policy should include urgent referral to a health team when a child is diagnosed with [an FASD]. Early intervention from an allied health perspective; the better the diagnosis and the better the outcome for the child with [an FASD]. So, I would definitely want that in policy. **(DOH**_**3**_ **Health professional**_**6**_**)**

However, other participants said there are many other policies that can address the issues experienced by individuals with FASD. They argued that the most important issue is the lack of proper implementation of existing policies because of limited resources.

I am adding to what participant eleven has said, there are lots of policies already [though] not specific to FASD but offered services for the children in general. The big concern is not the needs of the child [with an FASD] but lack of funding for the workforce **(DOH**_**4**_ **Health professional**_**10**_**)**

### Category 3: Current practices and available interventions

This category presents current practices and interventions for the prevention and management of FASD as reported by the service providers. The participants reported prevention and management interventions currently applied in their various organizations based on the clauses of FASD policies that exist in other South Africa policy documents. A list of the current practices and interventions for the prevention and management of FASD identified by the study participants are presented in Additional file 2.

The participants shared that they conduct educational programs in schools aimed at creating awareness for children on FASD. These programs usually focus on prevention, which the service providers do through experiential learning; making the children aware of the danger of drinking alcohol during pregnancy.

We have awareness programs in primary and high schools; focused on the prevention and early interventions. So, we have different programs in schools … **(DSD**_**2**_ **NPO workers**_**1**_**)**

We have a programme, which is called “Care for Babies.” It is of experiential learning to the primary learners’ (grade 6 and 7); experiential learning of foetal alcohol syndrome. We introduced fetal alcohol syndrome [FASD] at their levels. We do activities like playing games and symbolic talking message for children to understand what is fetal alcohol syndrome, the dangers and disabilities if a mom [mother] drinks alcohol during pregnancy. **(DSD**_**2**_ **NPO worker**_**2**_**)**

A participant reported on an intervention targeting adults (especially pregnant women) in the community. The intervention focuses on the effects of drinking alcohol during pregnancy.

We work in the community with the adult pregnant women; educate, inform, and support them. We share with them the effects of drinking alcohol during pregnancy. So, we have training sessions each month focusing on fetal alcohol syndrome, drugs, and pregnancy itself. We have individual sessions, motivational intervention talk, family sessions, group sessions, and pregnant women sessions. **(DSD**_**2**_ **NPO workers**_**1**_**)**

The participants discussed various interventions targeting pregnant women as they are considered the primary target for prevention.

We have two community workers working in two different areas. We will recruit 40 mentors and 120 pregnant women and divide them into two groups. We have 60 pregnant women and 20 mentors in one area and 60 pregnant women and 20 mentors in the other area. So, each mentor has to mentor three pregnant women and each community worker has to work with 20 mentors. **(DSD**_**2**_ **NPO workers**_**1**_**)**

We have a prevention program, where we work with pregnant women. We will recruit them before twenty weeks of the pregnancy and work with them through the pregnancy period. **(DSD**_**1**_ **NPO worker**_**4**_**)**

The participants mentioned other interventions that cover various segments of the community such as the substance abuse programme, which aims to raise awareness on FASD. Also, training of volunteers in the community to disseminate information on FASD to the community.

The Department of Social Development (DSD) has a programme, which is called substance abuse program and part of the substance abuse program is awareness intervention. However, they [DSD] do not deliver these services directly they use a service provider in NPOs to provide such services. **(DSD**_**2**_ **NPO worker**_**3**_**)**

We have a program called, Train the Trainer; we train social workers and volunteers (facilitators) in the community on fetal alcohol syndrome, alcohol, and drug abuse. So that they can now present fetal alcohol syndrome to the community and we give them visual presentation material that can be used to develop the community capacity on FASD information. **(DSD**_**2**_ **NPO workers**_**2&3**_**)**

Another participant shared services that focused on parents – counseling and educating on the effects of alcohol on the baby.

Our services focus on the parents, we counsel and educate about the impacts and effects of alcohol on the baby*.* **(DOH**_**2**_ **Health profesional**_**4**_**)**

Some participants reported the existence of referral systems for pregnant women drinking during their pregnancy and high-risk mothers. They mentioned the involvement of families and the provision of education, assistance, and referral aiming at helping the women to stop drinking.

When we see these pregnant patients [consuming alcohol], with dignity we take them to support groups to get assistance and we will involve their family where possible. We help high-risk mums, we get them help with delivery, and we will get involved during birth and post-delivery of the child. **(DOH**_**2**_ **Health professional**_**5**_**)**

When a mother has been identified consuming alcohol during pregnancy, then they [pregnancy women] will be referred to us. And we will do the education then refer [them] to the community, South African National Council on Alcoholism and Drug Dependence (SANCA), and all other organizations that are within the community that can assist the mother to stop drinking alcohol. **(DOH**_**2**_ **Health professional**_**1**_**)**

In addition to preventive approaches and interventions, the participants identified various management interventions that are currently applied in their organizations based on the FASD policy clauses existing in the different policies used for their organizational operation.

Some participants identified blanket interventions for every child that also encompassed FASD. Every child is screened for developmental delays including delays resulting from prenatal alcohol exposure.

Our service is part of [a] general intervention, as participants one and two have explained. We check every child for developmental delay, and if we identify the need for screening we will screen, and we will then follow through with the management as suggested by the result of the screening. **(DOH**_**2**_ **Health professional**_**8**_**)**

Some of the participants also reported allied health interventions for the comprehensive management of children with FASD.

We treat the FASD patients like any other patient, just like a child that is not speaking because the child has a language delay. We use the same strategies and techniques. **(DOH**_**2**_ **health professional**_**7**_**)**

We just do hearing screening for all children identified with maybe language delays to see if the delay is caused by hearing loss. **(DOH**_**2**_ **Health professional**_**8**_**)**

Some of the participants reported that proper diagnosis made at a tertiary hospital.

We refer them [the children] to be diagnosed at tertiary [hospital], and if required of us to follow up with the patient from a developmental perspective, we do [so] from when they [the children] are born to ages of six. **(DOH**_**2**_ **Health professioal**_**4**_**)**

In the clinic, we make a diagnosis, but from a genetic point of view, diagnosis requires [a] medical geneticist and further development follow-up is done at tertiary [hospital]. We will follow up with the patients as a team of allied health professional with the development of the patient. **(DOH**_**2**_ **Health professional**_**4**_**)**

Some of the participants indicated that a collaborative intervention involving the Department of Education’s office, the parents, and the schools towards assisting a child with learning disabilities (including FASD) should be established and coordinated.

We have psychologists and therapists working at the Department [of Education] so, a child can be referred there. For instance, if a child is in the mainstream and the child is struggling, they [psychologists and therapists] will assess and then refer [the child] to a school like us [special school]. **(DOE**_**3**_ **Teacher**_**5**_**)**

We have a very good relationship and partnership with the parents and we try to bring those [parents] on board to let them [parents] know what we do at all times. To let [parents] understand their children and for us to know what is going with the parents. We discovered these children have other social and family problems and that a lot of them are living in foster homes. **(DOE**_**3**_ **Teacher**_**4**_**)**

The participants shared interventions focused on educating children with FASD to the best of his/her ability. They indicated the provision of specialized and individualized education.

We give them [the children] an education that is our main purpose; they come here to be educated. This is a departmental school and according to white paper 6, every child has a right to be educated according to his/her ability. We educate them [the children] according to their ability – that is the intervention. We also do a sort of curriculum adaptation and extra curriculum activities such as sport and other things; that it is the service that we provide as a school. **(DOE**_**3**_ **Teacher**_**5**_**)**

We are dealing with SID (severe intellectual disabilities) and FASD are among them. When they [the children] are starting from preschool, we have an individualized educational plan [IEP], which is for each child in our classes. At the beginning of the year, occupational therapists and physiotherapist come in to do an assessment from which an IEP will be developed for each child and this will be followed for the year. **(DOE**_**3**_ **Teacher**_**1**_**)**

Some participants reported on schools available for educating children with FASD.

We are rendering residential care for children; specifically, children with FASD. We have Schools that accommodate the children that are affected with the FASD. **(DSD**_**1**_ **NPO worker**_**3**_**)**

Our school is a special school; all our children have a particular special need. So, each child is treated according to these special needs and the FASD children are not different in that sense. So, they have access to our education system. **(DOE**_**3**_ **Teacher**_**6**_**)**

A participant reported an adaption of the curriculum to make it functional for children with FASD.

Our curriculum is very much functional academic based. We take Curriculum and Assessment Policy Statements (CAPS) and make it more functional for our children. This is because if they are in our school they need to learn in a more practical way. **(DOE**_**3**_ **Teacher**_**4**_**)**

Some participants also reported that there were interventions that provided skills to individuals with FASD. They also identified a school that is providing skills to individuals with FASD between ages 14 to 18 years.

We provide social skills for them [individuals with FASD] so that they are socially developed in our society. We are teaching the children what they should be doing and how they should behave in society. **(DOE**_**3**_ **Teacher**_**4**_**)**

There are schools of skills, but they take children only from 14 to 18 and those are children who are usually stronger than the children in this type of school. So, some of them have FAS, I taught one of them [an individual with FASD] for some time in the Northern part of the country and she is doing very nicely. **(DOE**_**3**_ **Teacher**_**9**_**)**

Another participant described how individuals with FASD are provided with life skills.

We interact with the community in a sense; all the children are placed in a workshop environment or a work environment so that when they leave, they are equipped with lots life skills. **(DOE**_**3**_ **Teacher**_**6**_**)**

Participants re-counted various support structures for children, families, pregnant women, and individuals affected by FASD such as the school health system, which caters to children health needs when they are in school.

The school health system connects with the hospital, and social service[s] are involved right from the start as soon as there are concerns about alcohol use in pregnancy. **(DOH**_**2**_ **Health professional**_**4**_**)**

From the age of six, they [children] go to the school health clinic concerning health problems, so they [children] are followed up by the school health clinic as they are in school. And so, the adolescent is meant to be followed up by the school health system. **(DOH**_**2**_ **Health professional**_**4**_**)**

Some of the participants reported training of professionals such as teachers, social workers, psychologists, and other professionals as current programs in place.

We do professional training, where we speak with different professionals such as social workers, nurses to inform them about the services we offer. **(DSD**_**1**_ **NPO worker**_**3**_**)**

We also train teachers who work with the children, so that those teachers can look after/help the children in the class. So that the children do not fall too behind.We also work with the school psychologist in the areas, so that if the child is not coping with the mainstream school, they [psychologists] can refer the child to the special need schools. **(DSD**_**1**_ **NPO workers**_**4**_**)**

### Category 4: Identified policy requirements for FASD

This category presents the suggestions made by the participants on what they thought should be included in the FASD guideline. A table identifying all the interventions and programs that were suggested by the participants is submitted as Additional file 3.

Participants suggested that preventive awareness programs should be a continuous programme and should target people of all ages.

If they [government] would have better prevention awareness programs running, people would be aware of the secondary disability and its impact. **(DSD**_**1**_ **NPO workers**_**1**_**)**

So, I think the prevention should not actually start when they are already pregnant. I think that prevention should be aimed at the adolescent, to teach them good social drinking behaviours. **(DOH**_**3**_ **Health Professional**_**8**_**)**

Some participants suggested the need to include programs in the policy that target adolescents. The participants stated that adolescent should be well informed of the dangers of drinking alcohol during pregnancy.

We need to target the adolescent population, this is not like a health problem; it is a social/education problem. Once a mother is drinking during pregnancy, there is nothing you can do to change… **(DOH**_**3**_ **Health professional**_**4**_**)**

These days, teenagers begin to have sex and drinking alcohol at age of 13 years. So, I think even from primary school, children should be made aware of the negative effects of everything. **(DOH**_**3**_ **Health professional**_**8**_**)**

Participants also strongly emphasized the need for on-going social marketing by relevant mass media operators regarding sending messages to encourage pregnant women to refrain from drinking during pregnancy. The participants suggested the use of community leaders for social marketing.

Social development can put posters in the community for the community to connect with the media, adverts on TV, and radios. There is a lot of stuff that the social development can do as a department. **(DSD**_**1**_ **NPO worker**_**3**_**)**

There is a need for awareness, where this campaign is going out there and say do not drink when you are pregnant. They might use community leaders; they could be trained just in the basic information that drinking is not allowed for pregnant women. **(DSD**_**1**_ **NPO worker**_**1**_**)**

Participants proposed the need for awareness through social media including posters, awareness campaigns, and advertisements.

There is need to be marketing material; we need to use media that is impactful in the community. We should have a TV media programme in the community. **(DSD**_**1**_ **NPO worker**_**1**_**)**

On FASD awareness day (9 September), promoting public awareness is very important in schools, in health services, and in social work services. The government should have policies in place so that they can actively promote public awareness. **(DOE**_**3**_ **Teacher**_**6**_**)**

Participants suggested that there should be awareness at schools (primary and high) and University that no amount of alcohol is safe during pregnancy.

Prevention needs to be happening in the schools, teaching primary and high schools; bring intervention to them, then the Department of Health has Community Health Centres (CHC) to give posters and videos. **(DOH**_**3**_ **Health professional**_**5**_**)**

Going to the mainstream schools, high schools and telling people about FASD, training and letting them know about drinking during pregnancy. This is because many young people think that one or two drinks are fine to drink when you are pregnant, but they are not always aware of the dangers of drinking. Training young girls at university and letting them be aware. **(DOE**_**3**_ **SP**_**4**_**)**

A participant proposed the need for robust referral pathways for prevention to be included in the policy.

They [drinking women] need to be referred to medical professionals because once you took one child away from drinking women; the women need to be monitored. So, if these women become pregnant again, they must be urgently referred to an organization that already has a successful program running, so that intervention can take place. **(DSD**_**1**_ **NPO worker**_**1**_**)**

The participants proposed that the problems associated with FASD could not be resolved by one department. Thereby the participants suggested that the policy should facilitate collaboration for preventive efforts among departments.

We need to start providing early intervention from the beginning. There must be a multidisciplinary group in place immediately around the child. So, policy should be or need to state what will happen with the child. **(DSD**_**1**_ **NPO worker**_**3**_**)**

It is not one specific department, which is supposed to be in charge of this [prevention]. We need to start with the young children so that we can prevent it from happening again in the future. **(DOH**_**3**_ **Health Professional**_**8**_**)**

Some participants suggested that the policy should facilitate training and support for parents and caregivers of children with FASD to understand the challenges faced by children and learn skills to help them better manage.

A support system may be for the parents; they [parent] should be involved in workshops on how to treat and raise children with FASD. This is because I am sure that they [parent] come from the streets and a lot [of] them do not understand what is wrong with their child. **(DSD**_**2**_ **NPO worker**_**1**_**)**

They [professionals] need to train these parents on the primary and secondary disabilities [related to FASD]. There is a need for the social workers to go to these houses to provide emotional support for the parents and make sure that the children are in caring homes. **(DSD**_**1**_ **NPO worker**_**3**_**)**

Some participants identified the need for a separate school and revitalisation of special classes in the mainstream school for children with FASD. The reason the participants provided was that children with FASD might feel inferior to other children because of their intellectual abilities.

They [children with FASD] need to be separated from the normal children, so I think we need to build the schools for FASD children. But, we take the same school curriculum and adapted it to suit their needs and ability. They [children with FASD] might feel inferior to other children because they cannot do what other children can do. **(DSD**_**2**_ **NPO worker**_**2**_**)**

We need more [special] schools like this, maybe smaller ones in each smaller community but the school that will cater for the needs of these children at the different levels. Also, they [government] should bring back those special classes to the mainstream schools. **(DOE**_**3**_ **Teacher**_**9**_**)**

Some of the participants advocated that policy should acknowledge that FASD is multiprofessional and interdepartmental issue and the departments should collaborate in addressing the gaps around FASD services.

I think the policy should encourage corporation between [Departments of] Education, Health, and Social Development; they should work together. It should be put into the policy that health services should support the education and social services. (**DOE**_**3**_ **Teacher**_**6**_**)**

One department cannot do this in isolation, there should be a fundamental process of diagnosis. When a child with an FASD is diagnosed by the Department of Health, then the Department of Social Development should be informed so that they can follow up with the child. **(DSD**_**1**_ **NPO worker**_**2**_**)**

A participant proposed the need for a referral pathway for management to be included in the policy so that better outcomes can be achieved for individuals with FASD.

The policy should also include urgent referral to a health team when a child is diagnosed. The earlier the intervention from allied health professionals, the better the prognosis and the outcome for the child. So, I would definitely want that in policy, the children should be referred early. **(DOH**_**3**_ **Health Professional**_**6**_**)**

Some participants want courses and training on how to manage FASD to be included in medical training as this is currently absent. Participants were of the opinion that policy could facilitate this.

You never get the training or given a skill on how to manage these [FASD]. You were just given information about FASD. So, you were not given the skill after the training that can be used to manage these kinds of cases (FASD) **(DOH**_**3**_ **Health Professional**_**1**_**)**

We do not receive a specific course on FASD, we would just manage the patients based on the symptoms they present. We will manage FASD children like any other child with developmental delay. **(DOH**_**3**_ **Health Professional**_**6**_**)**

A participant proposed training of professionals such as lawyers, teachers, social workers, and nurses to enable a proper understanding of FASD issues.

There should be training for the professionals, lawyers, teachers, social workers, and nursing staff; so that they could start to recognize what is actually happening with individuals with FASD. **(DSD**_**1**_ **NPO worker**_**1**_**)**

The participants reported that they were given generalized training on disabilities as a special need for teachers, and reported a lack of specific training on FASD. The participants thus suggested that policy could facilitate specialized training on FASD; especially training on how to identify affected children and how to counsel the parents of these children.

We are all trained as special needs teachers but there is no specific training for FASD. We are special needs teachers, we cover every diagnosis [disability] that children may have. They [trainer] are not saying there is a special training for FASD. **(DOE**_**3**_ **Teacher**_**1**_**)**

The Department [of Education] organises many workshops but we do not have workshops specifically on FASD. So, the Department must provide a way for us on how to identify children and an on-going training on screening potential children. Also, training on how to counsel the parent because parents are the reason why the children are [suffering from] FASD as they [the parent] abused alcohol during pregnancy. **(DOE**_**3**_ **Teacher**_**6**_**)**

## Discussion

This study is part of a project aimed at designing a guideline to inform policies on FASD [35]. In this study, we explored the perspectives of different service providers, regarding various existing and required preventive and management approaches for FASD. Additionally, those strategies that could facilitate a multidisciplinary, multi-sectoral, and holistic approach to FASD.

The manifestations of FASD can involve primary and secondary disabilities [52]. These disabilities usually include educational, medical, and social issues. At present, each department in a generic manner addresses the issues related to FASD based on the clauses that exist in their current policies and guidelines. For example, the Department of Education mostly focuses on addressing educational issues (learning disabilities). These are not particularly related to, but may include FASD, and are not particularly focused on the broader medical and social issues. Similarly, the Departments of Health and Social Development, follow policies that address general health issues and social problems, respectively.

With each department delivering care for individuals with FASD working ‘in silos’ and following a nonspecific approach from their own perspective, the prevention and management of FASD remains uncoordinated and ineffective. This approach is contrary to the multidisciplinary, multisectoral and holistic approach required for the proper management of FASD [53], as FASD-related issues are multifaceted with intrinsic and extrinsic factors responsible for the outcomes. Clauses relevant to the prevention and management of FASD that exist in other policies have not been able to effectively address the issues of FASD, which could be facilitated by the creation of a specific FASD policy [11, 54].

Despite that there are various current preventive interventions reported, the prevalence of FASD in South Africa, especially in the Western Cape Province continues to be high [15]. Therefore, researchers and public health specialists advocate the need for preventive interventions that are sensitive, responsive, and appropriate to the context [11, 54]. In South Africa, this is an indication that the current preventive interventions have not been effective in reducing the prevalence of FASD.

The reasons for the persistently high prevalence of FASD could include the generic nature of interventions for FASD prevention and management. Not only that, but it could also be the current lack of specific prevention interventions for women of reproductive age and their partners or families. Of which, the latter is level-two of four levels of prevention for FASD as proposed by Poole et al. [22]. The reason being that the alcohol exposure is likely to occur before women know they are pregnant and therefore before they present for prenatal care. Therefore, prevention needs to be targeted more broadly and require proper understanding of alcohol and contraceptive use and norms of reproductive age [31].

Furthermore, the current prevention approaches are focused on educating people about the risks of drinking during pregnancy without taking into account the wider societal problems with alcohol use in the community. These approaches are assuming that the community just needs to be provided with expert information about the risks of alcohol consumption and they will be able to change their behavior. However, research from other drug/alcohol areas show that education does not necessarily change behavior [55, 56]. Therefore, policy needs to address the fact that prevention of alcohol use during pregnancy is much more complicated than just providing information about the risks of drinking during pregnancy. Also, policy needs to address factors that influence women’s drinking such as having partners or family members who drink and experiencing domestic violence, as research has shown women experiencing domestic violence are more likely to abuse alcohol [57].

In addition to the above reasons, the high prevalence of FASD in South Africa could be attributed to a lack of re-contextualization and decolonization of current policy discourse and failure to reframe the problem of FASD. Thus, the solution to the FASD problem could be realized by addressing the structural, social and historical processes such as urbanization, racialization, and colonialism that are responsible for women’s health and social inequities [58]. Moreover, most of the current interventions are not evidence-based as there is a paucity of local research in South Africa [59]. Therefore, there is a need for specialized and targeted preventive services and the need to address gaps in current policies and policy implementation to facilitate effective intervention [20, 32].

In a broader perspective, the reason for ineffective interventions could also be related to the characteristics of the communities [14], which are marginalized environments where the pre-requisites of health (peace, food, shelter, sustainable income/livelihood, and stable ecosystem) are not met. Therefore, there is the need to address the above social determinants of health that pre-conditioned the health of these individuals. For this reason, we are developing guidelines to facilitate a holistic policy for FASD, which entails the participation of all in the prevention and management of FASD.

The ineffectiveness of the current preventive interventions could also be a result of the barriers identified previously in Canada [22]. These include the conflicting messages in the media about the amount of alcohol that can be consumed during pregnancy and lack of skills by service providers to discuss alcohol with women and their inability to connect women to the appropriate services. Women also fear losing custody of their children if they access services and existing barriers to accessing support systems such as lack of affordable housing and transportation [22]. Therefore, in addressing these barriers to effective interventions, a policy is needed.

We noted that there are available management services, however only few specifically target FASD [31]. We also discovered that there are only few management services targeting adults with FASD [60]. Our study highlights the need for a specialized intervention, which will enable children with FASD to receive early intervention and follow-up to adulthood [61, 62]. Early interventions show promising outcomes and have the ability to prevent secondary disabilities. Therefore, to facilitate appropriate interventions at every age, policy is needed, as the effective management of primary and secondary disabilities require sustainable commitment from government and service providers [18, 21].

We found various suggestions on what could be included in a guideline/policy for the prevention and management of FASD in South Africa. Some of our findings were supported by other studies that had examined the policy and intervention requirements for the prevention and management of FASD, locally [31, 63] and internationally [30, 64, 65]. These findings include support for individuals with FASD and their families, public education and awareness, specialized support for pregnant women, training for relevant professionals and community awareness of FASD to prevent prenatal exposure to alcohol. Therefore, local and international research could assist in informing policy development in South Africa.

Locally, Rendall-Mkosi et al. [31] identified service coordination and consensus building, surveillance and detection of alcohol-exposed pregnancy (AEP) and FASD, monitoring alcohol use in women of childbearing age, the detection of and intervention with women at risk of AEP, public education and awareness, and intervention and support for individuals with FASD. The above interventions were seen as efforts that could facilitate the prevention and management of FASD. In a policy brief in 2008, policy requirements considered to address FASD issues included surveillance and monitoring, screening and brief interventions for women, awareness raising and education on FASD, liquor control, and research gaps in services and interventions [63].

Internationally, Canadian experts have identified a four-level model for FASD prevention [65]. This includes public awareness and broad health promotion, conversations about alcohol with women of childbearing age and their partners, specialized support for pregnant women and postpartum support for new mothers [65]. The above interventions were similar to our findings on preventive intervention requirements for guideline/policy. George and Hardy [64] analyzed the funding proposals submitted on FASD in Canada and identified the following proposed strategies for the management of FASD:

1. Support for individuals with FASD and their families to develop life, social, parenting, and employment skills;
2. Access to direct services such as housing and care in schools;
3. Intensive training for professionals working with individuals with FASD and the community;
4. Provision of libraries and resource rooms, curriculum, programme manuals on FASD;
5. Access to training designed to raise awareness about FASD;
6. Support for individuals with FASD and their families during significant life transitions;
7. Peer support for children and youth with FASD and their parents;
8. Research on interventions targeting various FASD outcomes; and
9. Diagnostic clinics for adults and the creation of networks of professionals and families.

The Australian Action Plan, in which five priority areas for the prevention and management of FASD across the lifespan were identified, resembled those found by George and Hardy [64]. These included increasing community awareness of FASD to prevent the prenatal exposure to alcohol and improve the diagnostic capacity of FASD. Furthermore, interventions to assist individuals with FASD to achieve their full potential, improve data collection to understand the extent of FASD, and close the gap on the high prevalence among certain population were recommended [30].

Findings from this study have significant public health implications. The fact that the current practices have not reduced the prevalence of FASD in South Africa should be seen as a challenge to public health. Given that many women are aware of the dangers of drinking alcohol during pregnancy, the prevalence is still high showing that we need to reconsider how we are approaching the prevention of the problem. Efforts should be made to urgently address the maternal risk factors for FASD [42] and the social determinants that precondition the behavior and health of these women [14]. In addition, we recommend that further research is needed to evaluate current practices and interventions systematically, to see the extent they have prevented FASD or benefited the individuals with FASD. Further research is also needed to investigate the barriers and facilitators to reducing the prevalence of FASD is South Africa.

### Strengths and limitations of the study

In this study, we used focus groups as a method of inquiry, which allowed the researchers to get a response from multiple participants. Furthermore, it allowed participants to validate each other’s responses. A limitation of this study is that we recruited available participants in a single setting for most of the focus groups, as it was difficult to combine participants from different settings. This prevented some professionals from participating in the focus group discussions thus limiting the variations in the type of participants per focus group. Furthermore, the participants from the Department of Labour and Department of Justice who supposedly render services to individuals with FASD were not sampled, while their responses might be valuable in developing an integrated policy.

## Conclusion

Despite various prevention and management strategies being currently implemented, the prevalence of FASD remain high in South Africa, especially in the Western Cape Province. The reason for the persistently high prevalence of FASD could be related to the generic nature of FASD prevention and management approaches, lack of evidence-based approaches and coordinated effort. Therefore, the need for a policy to ensure a coordinated and holistic effort for the prevention and management of FASD is highlighted.

### Additional files

Additional file 1: Interview guide for focus groups interviews. (DOCX 14 kb)

Additional file 2: Current practices and interventions. (DOCX 19 kb)

Additional file 3: Identified policy requirements. (DOCX 18 kb)

## Appendix 1

**Table 3:** Coding guide

Categories	Themes	Sub-themes
Availability (lack) of policies/ guidelines on FASD	No specific guideline/policy document on FASD	
	Clauses in other guidelines/policy documents	
Development of guideline/policy document	There is a need to develop separate guideline/policy document	
	There is no need to develop separate guideline/policy document	
Current practices and available interventions	Prevention-related interventions	Clinical prevention interventions
		Educational prevention interventions
		Social prevention interventions
	Management-related interventions	Clinical management interventions
		Educational management interventions
		Social management Interventions
Identified policy requirements for FASD	Prevention-related interventions	Clinical prevention policy requirements
		Educational prevention policy requirements
		Social prevention policy requirements
	Management-related interventions	Clinical management policy requirements
		Educational management policy requirements
		Social management policy requirements

## Appendix 2

**Table 4:** Exploring service providers’ perspectives on the prevention and management of fetal alcohol spectrum disorders in South Africa: A qualitative study

No. Item	Guide questions/description	Reported on Page #
Domain 1: Research team and reflexivity		
*Personal Characteristics*		
1. Interviewer/facilitator	Which author/s conducted the interview or focus group?	Results (5) Babatope O. Adebiyi
2. Credentials	What were the researcher’s credentials? E.g. PhD, MD	Babatope O. Adebiyi PhD Student Ferdinand C. Mukumbang PhD Lizahn G. Cloete PhD Anna-Marie Beytell PhD
3. Occupation	What was their occupation at the time of the study?	Student, Post-Doctoral researcher, Lecturer, Lecturer
4. Gender	Was the researcher male or female?	Male, Male, Female, Female
5. Experience and training	What experience or training did the researcher have?	Methods (5) They all have extensive experience in conducting qualitative study
*Relationship with participants*		
6. Relationship established	Was a relationship established prior to study commencement?	(4) Yes
7. Participant knowledge of the interviewer	What did the participants know about the researcher? e.g. personal goals, reasons for doing the research	(4) Participant information sheet and Consent form
8. Interviewer characteristics	What characteristics were reported about the inter viewer/facilitator? e.g. Bias, assumptions, reasons and interests in the research topic	Methods (5) The participants know that the primary researcher is a student
Domain 2: study design		
*Theoretical framework*		
9. Methodological orientation and Theory	What methodological orientation was stated to underpin the study? e.g. grounded theory, discourse analysis, ethnography, phenomenology, content analysis	Methods (4, 5) – Exploratory qualitative design – Framework Method
*Participant selection*		
10. Sampling	How were participants selected? e.g. purposive, convenience, consecutive, snowball	Methods (4) Purposive sampling
11. Method of approach	How were participants approached? e.g. face-to-face, telephone, mail, email	Methods (4) face-to-face, telephone, email
12. Sample size	How many participants were in the study?	Results (4) 65
13. Non-participation	How many people refused to participate or dropped out? Reasons?	Methods (4) 3 (It was because of the work load)
*Setting*		
14. Setting of data collection	Where was the data collected? e.g. home, clinic, workplace	Methods (5) Clinic, workplace
15. Presence of non-participants	Was anyone else present besides the participants and researchers?	Results (5) No
16. Description of sample	What are the important characteristics of the sample? e.g. demographic data, date	Results (4) Profession, Gender and Working experience
*Data collection*		
17. Interview guide	Were questions, prompts, guides provided by the authors? Was it pilot tested?	Methods (5) No
18. Repeat interviews	Were repeat interviews carried out? If yes, how many?	N/A
19. Audio/visual recording	Did the research use audio or visual recording to collect the data?	Methods (5) Yes
20. Field notes	Were field notes made during and/or after the interview or focus group?	Methods (5) Yes
21. Duration	What was the duration of the inter views or focus group?	Methods (5) 30–60 min
22. Data saturation	Was data saturation discussed?	Methods (5) Yes
23. Transcripts returned	Were transcripts returned to participants for comment and/or correction?	N/A No
Domain 3: analysis and findings		
*Data analysis*		
24. Number of data coders	How many data coders coded the data?	Methods (5) 2
25. Description of the coding tree	Did authors provide a description of the coding tree?	No
26. Derivation of themes	Were themes identified in advance or derived from the data?	Methods (5) Derived from the data?
27. Software	What software, if applicable, was used to manage the data?	N/A
28. Participant checking	Did participants provide feedback on the findings?	Strengths and limitations No
*Reporting*		
29. Quotations presented	Were participant quotations presented to illustrate the themes/findings? Was each quotation identified? e.g. participant number	Results (5–11) Yes
30. Data and findings consistent	Was there consistency between the data presented and the findings?	Result and Discussion (5–13) Relationship to existing knowledge Yes
31. Clarity of major themes	Were major themes clearly presented in the findings?	Results (5–11) Yes
32. Clarity of minor themes	Is there a description of diverse cases or discussion of minor themes?	Discussion (11–13) Yes

## Abbreviations

AEP: Alcohol-exposed pregnancy
CAPS: Curriculum and assessment policy statements
CHC: Community Health Centre
COREQ: Consolidated criteria for reporting qualitative research
DOE: Department of Education
DOH: Department of Health
DSD: Department of Social Development
FASD: Fetal alcohol spectrum disorders
FGD: Focus group discussions
IEP: Individualized educational plan
IQ: Intelligence quotient
NPO: Non-profit Organisation
SANCA: South African National Council on Alcoholism and Drug Dependence
SAPS: South Africa police service
SID: Severe intellectual disabilities
TV: Television
UIF: Unemployment insurance fund
USA: United State of America
