# Supplementary material for: Exploring service providers’ perspectives on the prevention and management of fetal alcohol spectrum disorders in South Africa: a qualitative study
Source: BMC Public Health. 2018 Nov 6;18:1238. doi: 10.1186/s12889-018-6126-x (PMC6220472; doi:10.1186/s12889-018-6126-x)
Supplement: Supplementary file 1 — Interview guide for focus groups interviews. (DOCX 14 kb) [file 12889_2018_6126_MOESM1_ESM.docx]

**INTERVIEW GUIDE FOR FOCUS GROUPS INTERVIEWS:**

**Introductions and purpose of interview explained and consent form signed.**

1. Which services and interventions do you provide to individuals with FASD?
2. In what context – as part of policy or general interventions?

**Themes to discuss or probe**:

**Department of Health:**

- Diagnosis, follow-up services after diagnosis
- Ages and development (what about adolescents and adults)?
- Mental health and psychiatric conditions
- Use of alcohol/substances
- Sexual behavior
- Preventative services = mother alcohol use, health promotion maternal, and community education

**Department of Education:**

- School experience of individuals with FASD
- Absence from school
- Specific language, cognitive, literacy, social skills or other training? (Child and parents)
- Parental involvement
- Ages and development (what about adolescents)?

**Department of Social Development**

- Continuing services to individuals with FASD irrespective of age
- Family services and single parent services
- Living conditions and recreational facilities
- Employment and recreational activities
- Inclusion in communities, schools etc.
- Financial assistance
- Disability services
- Alcohol and substance abuse
- Sexual behavior
- Conflict with law

Preventative services: alcohol abuse of mothers, communities, alcohol as payment, social skills training, and family coping with FASD

1. Describe the services that are currently provided by your department to individuals living with FASD, families and community?
2. Can you please describe the type of services you think your department should be providing for individuals living with FASD, families, and community?
3. Describe which services/interventions do you think should be included in policy or guidelines for practice in terms of individuals with FASD, families, caregivers, communities, and service providers (training, resources and collaboration between departments)? How can they be included?
4. In developing guidelines that will inform policy development express your opinion on those things you think should be included/excluded in the guidelines?
5. Describe how adequate are the responses of your department to FASD with regards to existing policies/guidelines and services?
