# Supplementary material for: Exploring service providers’ perspectives on the prevention and management of fetal alcohol spectrum disorders in South Africa: a qualitative study
Source: BMC Public Health. 2018 Nov 6;18:1238. doi: 10.1186/s12889-018-6126-x (PMC6220472; doi:10.1186/s12889-018-6126-x)
Supplement: Supplementary file 2 — Current practices and interventions. (DOCX 19 kb) [file 12889_2018_6126_MOESM2_ESM.docx]

**Additional File 2:** shows the different practices, services, interventions, and programmes that are currently in place according to the participants for the prevention and management of FASD.

***Additional file 2:* *Current practices and interventions***

| Prevention practices and interventions | Clinical | Referral to Non-profit Organisations with service for people with alcohol problem |
| --- | --- | --- |
|  |  | Advising pregnant women on alcohol use |
|  |  | Taking alcohol history during antenatal booking |
|  |  | Counselling and referral to the community-based organisations and support group for assistance |
|  |  | Alcoholic women referred to Social workers |
|  |  | Family involvement for at-risk mothers |
|  | **Educational** | Prevention awareness in schools on alcohol |
|  |  | Community talks on food and alcohol use during pregnancy |
|  |  | Training of community workers to educate communities on FASD |
|  |  | Distribution of information pamphlets on alcohol and alcohol abuse |
|  |  | Health education for women |
|  |  | Train the trainers programme on FASD |
|  | **Social** | Support for women in the community to abstain from alcohol during pregnancy. |
|  |  | Including pregnant women in identifying programmes to prevent FASD |
|  |  | Raising awareness through theatre, experiential learning, and night sessions in the community and schools |
|  |  | Educate, inform, mentor, and support pregnant women |
|  |  | Motivational counselling for pregnant women |
| Management practices and interventions | **Clinical** | Screening, diagnostic, assessment, and therapy for children with FASD |
|  |  | General developmental screening at six weeks for children |
|  |  | Assisting a child with language and feeding problem |
|  |  | Referral for a child with developmental delay to appropriate services |
|  |  | Developmental stimulation for children with FASD with developmental delay |
|  |  | Mental health service for children with FASD |
|  |  | General medical support for children with FASD |
|  |  | Interventions to improve fine and gross motor for children with FASD |
|  |  | Counselling and educating parents on a child diagnosis and management at home |
|  |  | Exercise intervention for children with FASD |
|  |  | General management plan for children with developmental delay |
|  |  | Referral of children with FASD to children homes |
|  | **Educational** | Educating children with FASD |
|  |  | Extracurricular activities for children with FASD |
|  |  | Language, mathematics, social and life skill development for children with FASD |
|  |  | Placing children with FASD in a workshop environment. |
|  |  | Development of IEP and curriculum based on assessment |
|  |  | Appropriate school referral for children with FASD |
|  |  | Availability of school of skill for older children |
|  |  | Parent involvement in management of children with FASD |
|  |  | School health clinic assist with the management of children at school |
|  |  | Occupational services aim to improve learners’ outcome |
|  |  | Storytelling to improve child cognitive |
|  |  | Exercise activities (finger, drawing, and painting) for children with FASD |
|  |  | Training of professionals and parents on FASD management |
|  |  | Special teacher and support teacher to assist the children with FASDs |
|  |  | Use of picture for explanation of concepts in the classroom |
|  |  | Early childhood schools for children with FASD |
|  | **Social** | Residential service for children with FASD |
|  |  | Interaction with community toward assisting a child with FASD |
|  |  | Advocating for the right of individuals with FASD |
|  |  | Availability of foster care for children with FASD |
|  |  | Support for parents’ with a child with FASD |
|  |  | Availability of protective workshops for adult with disability |
|  |  | Availability of daycare service for children with disability |
|  |  | Assistance with grants application |
