# Supplementary material for: Exploring service providers’ perspectives on the prevention and management of fetal alcohol spectrum disorders in South Africa: a qualitative study
Source: BMC Public Health. 2018 Nov 6;18:1238. doi: 10.1186/s12889-018-6126-x (PMC6220472; doi:10.1186/s12889-018-6126-x)
Supplement: Supplementary file 3 — Identified policy requirements. (DOCX 18 kb) [file 12889_2018_6126_MOESM3_ESM.docx]

**Additional file 3:** illustrates all the various practices, interventions, and programmes identified by the participants that could be probably included in a policy for the prevention and management of FASD.

***Additional file 3: Identified policy requirements***

| Prevention policy requirement | Clinic setting | Encourage early booking for pregnant women |
| --- | --- | --- |
|  |  | Counselling services for women on alcohol abuse |
|  |  | Holistic approach to FASD prevention |
|  |  | Refer and support for women with alcohol problems |
|  |  | On-going counselling on FASD in clinics |
|  |  | Use of community health forum for FASD prevention |
|  | **Educational setting** | Education on danger in drinking during pregnancy |
|  |  | Health education on FASD for all |
|  |  | Awareness in schools and public places on FASD |
|  |  | Poster in building on danger of drinking alcohol during pregnancy |
|  |  | Use of labelling, posters, adverts, pamphlets, and video on FAS for campaign |
|  |  | Education on FASD for teenagers and in schools |
|  |  | Prevention programme on effect of alcohol for schools and community |
|  |  | Use of billboard for messages on maternal drinking |
|  |  | Use peer education for prevention |
|  |  | Skill training for women in the community |
|  |  | Awareness of no amount of alcohol |
|  | **Social environment** | Working with other sectors like Police service |
|  |  | Regulation of shebeens/taverns to control alcohol sales especially to pregnant women |
|  |  | After school activities to promote healthy behaviours |
|  |  | Use of community-based workers to delivered message on FASD at homes in the community |
|  |  | Enforcing law against alcohol abuse |
|  |  | Community-based approach to FASD prevention |
|  |  | Use of law to prevent pregnant mother from buy alcohol |
|  |  | Use of community work to assist women |
| Management policy requirements | **Clinic setting** | Awareness and prevention of FASD in clinics |
|  |  | Motivational talk and counselling in clinics to reduce drinking |
|  |  | Integrating system of identification and coordination of services for children |
|  |  | Early diagnosis and management of FASD |
|  |  | Provision of mental health service to individuals with FASD |
|  |  | Stimulation of child’s development |
|  |  | Create national surveillance for FASD |
|  |  | Thorough developmental screening for children |
|  |  | Establishment of a multidisciplinary team for management of FASD |
|  |  | Clear referral pathway between departments on management of FASD |
|  |  | Development of appropriate age interventions for children with FASD |
|  | **Educational setting** | Interdepartmental involvement (SAPS, churches, DOE, DSD, DOH, and NPOs) |
|  |  | Training and workshops for teachers on how to counsel and support parents’ who have children with FASD |
|  |  | Educate parents and support groups on FASD management |
|  |  | Provision of specialised school for children with FASD |
|  |  | Refer children with FASD who are struggling in the main school to special school |
|  |  | Comprehensive learners’ bio-data for children with FASD |
|  |  | Involvement of the parents in management of children with FASD children |
|  |  | Assist learners with schools closer to their home |
|  |  | Increase government involvement in schools |
|  |  | Modification of CAPS and use of IEP for children with FASD |
|  |  | Advert on TV, radios, and posters on FASD in the community |
|  |  | Support for children with FASD in the mainstream |
|  |  | Building more special school |
|  |  | Training of social worker, health professionals, and teachers on FASD |
|  |  | Provide individuals with FASDs with various skills |
|  |  | Assist teachers with classroom management |
|  |  | Appropriate placement for learner ship for individuals with FASD |
|  | **Social environment** | Support system for individuals with FASD and family in the community and in schools |
|  |  | Provision of social grant for individuals with FASD |
|  |  | Support programmes for individuals with FASD in foster care |
|  |  | Development of proper implementation plan for policy on FASD |
|  |  | Awareness programme on primary and secondary disabilities of FASD in the community |
|  |  | Promote independent living for individuals with FASD |
|  |  | Community services aim at integrating individuals with FASD |
|  |  | Provision of grants in form of food to Individuals with alcohol problems |
|  |  | Awareness and prevention of FASD in the community |
|  |  | Protection for individuals with FASD |
|  |  | Build residential and day care facilities |
|  |  | Ban alcohol advertisement in media |
